# Multi-rotor imidazole-based fluorophores for rapid delineation of squamous cell carcinoma infiltration margins in murine surgical specimens

**DOI:** 10.3389/fchem.2026.1913502

**Published:** 2026-08-10

**Authors:** Huabin He

**Affiliations:** Department of Hand and Foot Surgery, Yiwu Central Hospital, Yiwu, Zhejiang, China

**Keywords:** aggregation-induced emission, intraoperative biopsy, squamous cell carcinoma, triphenylimidazole, tunable emission

## Abstract

Multi-rotor imidazole derivatives have attracted more and more attention due to their unique electronic and photophysical properties as well as diverse applications. Combined with aggregation-induced emission (AIE) mechanism, multi-rotor imidazole core could also be functionalized as AIE luminogens (AIEgens). Herein, we report a novel family of AIEgens, designated as TPIT-X, which features a donor-π-acceptor (D-π-A) backbone wherein 1,4,5-triphenylimidazole serves as the donor and thiophene acts as the π-linker. By introducing appropriate acceptor units, we designed and synthesized TPIT-1 to TPIT-6, which exhibit tunable emission colors ranging from green (490 nm) to deep red (695 nm), covering nearly the entire visible spectrum. These molecules also display typical AIE characteristics, with up to 30-fold fluorescence enhancement. Taking advantage of their AIE feature, TPIT-6 nanoparticles conjugated with anti-EGFR antibodies successfully realized rapid determination of squamous cell carcinoma (SCC) tumor infiltration margins in *ex vivo* mouse surgical specimens, thereby positioning them as promising imaging agents for rapid intraoperative tumor biopsy during hand SCC resection.

## Introduction

From a clinical standpoint, precise resection of hand tumors is of great importance since hands serve vital functions in everyday activities. Even small hand lesions can give rise to ongoing pain, noticeable deformities, or functional deficits, greatly diminishing patients’ quality of life. Among hand tumors, the most frequently encountered malignancy is squamous cell carcinoma (SCC), which accounts for 58%–90% of total cases (Mavrogenis et al., 2017; Valk et al., 2020). Given that surgical resection remains the mainstay of treatment for hand SCC, real-time intraoperative identification of SCC tumor margins is of critical significance. Overly aggressive resection may impair both the appearance and essential functions of the hand, whereas excessively conservative resection carries the risk of leaving residual tumor tissue. These retained tumor cells can subsequently invade adjacent structures, particularly regional lymph nodes. According to published data, approximately 14.3% of SCC patients develop distant metastases within 5 years, which is associated with worsened prognosis (Caud et al., 2023; Sayed et al., 2019). Current intraoperative assessment of resection margins mainly relies on frozen section analysis on selected tissue specimens, which involves a time-consuming multistep process (specimen collection, transportation, freezing, sectioning, staining, and pathological interpretation). This technique only provides information for a limited number of specimens, and its accuracy is highly dependent on the pathologist’s experience to distinguish cellular morphology. Furthermore, the formation of ice crystals during freezing may impair cellular integrity, thereby affecting accurate cell identification. Therefore, researchers worldwide have been exploring various alternative techniques for the real-time assessment of tumor margins.

Fluorescence-based approaches are proved to be excellent candidates for such applications owing to their high sensitivity and easy detectability (Refaat et al., 2022; Shcheslavskiy et al., 2025; Wang et al., 2024). Despite the abundance of fluorescent molecules, most traditional organic fluorophores always suffer from aggregation-caused quenching (ACQ) effect, which is characterized as emission diminishing or even complete quenching in aggregated states, severely restricting their utilizations in many cases. First proposed in 2001, the concept of aggregation-induced emission (AIE) has greatly reformed the conventional rules of fluorophore development. In contrast to ACQ fluorophores, aggregation-induced emission luminogens (AIEgens) exhibit weak or no emission in a dispersed state but become significantly luminescent upon molecular aggregation. This unique feature enables AIEgens as revolutionary materials for biosensing and bioimaging. Thus AIE was selected as the 2020 top ten emerging technologies in chemistry by IUPAC (Jiang et al., 2024; Luo et al., 2001; Mei et al., 2015). Although a wide variety of AIEgens have been reported to date, the development of systems with finely tunable emission wavelengths to meet diverse application demands remains highly significant (Kothavale and Sekar, 2017; Song et al., 2016; Xu et al., 2019). In this study, we conceived and developed a novel emission-tunable AIE platform based on a 1,4,5-triphenylimidazole core structure. This newly established platform, designated TPIT-X, features a donor–π–acceptor (D–π–A) backbone in which thiophene serves as an electron-rich π-linker. By simply introducing appropriate acceptor units, we synthesized a series of AIE emitters (TPIP-1 to TPIP-6) that exhibit broad emission windows ranging from green (490 nm) to deep red (695 nm), thereby covering almost the entire visible spectrum. Optical evaluations also confirmed their pronounced AIE characteristics. As a highly hopeful application method for AIE fluorophore, fluorescence-guided surgery (FGS) has been extensively studied in previous reports: following systemic administration of targeted contrast agents, the tumor region becomess highly luminescent. Surgeons can then either directly resect additional diseased tissue under fluorescence guidance or collect suspicious margin samples for further evaluation via paraffin section analysis. Inspired by these findings, we recognize that the direct application of fluorescence imaging in intraoperative pathological diagnosis is both promising and attractive, owing to its operational simplicity, rapid turnaround time and non-invasive nature, which largely circumvents the risk of adverse reactions. Importantly, in contrast to injectable fluorescent probes used in FGS, contrast agents employed for *ex vivo* detection are classified as Class III medical devices and do not require drug approval. Consequently, they can achieve faster market authorization with lower development risks. In consideration of the inherent superiority of AIE molecules with deep red emission for bioimaging, TPIT-6 was encapsulated with DSPE-PEG2000 and further conjugated with Anti epidermal growth factor receptor (Anti-EGFR) antibodies to prepare nanoparticles (NPs)-based fluorescent probes. Based on TPIT-6 NPs-Ab, we designed a fluorescence imaging-based method for rapid visualization of tumor margins, aiming to overcome the challenges that impede real-time pathological diagnosis during SCC surgery. In our workflow, freshly excised skin tissue samples were co-incubated with the EGFR-targeted TPIT-6 NPs-Ab fluorescent probe. This approach successfully enabled rapid localization of tumor boundaries within the tissue specimens under fluorescence imaging. In brief, our work not only establishes an innovative design strategy for AIEgens with wide color tunability but also provides a novel intraoperative pathological analysis technique for hand SCC tumor resection.

## Results and discussion

### Design and synthesis

**SCHEME 1:**
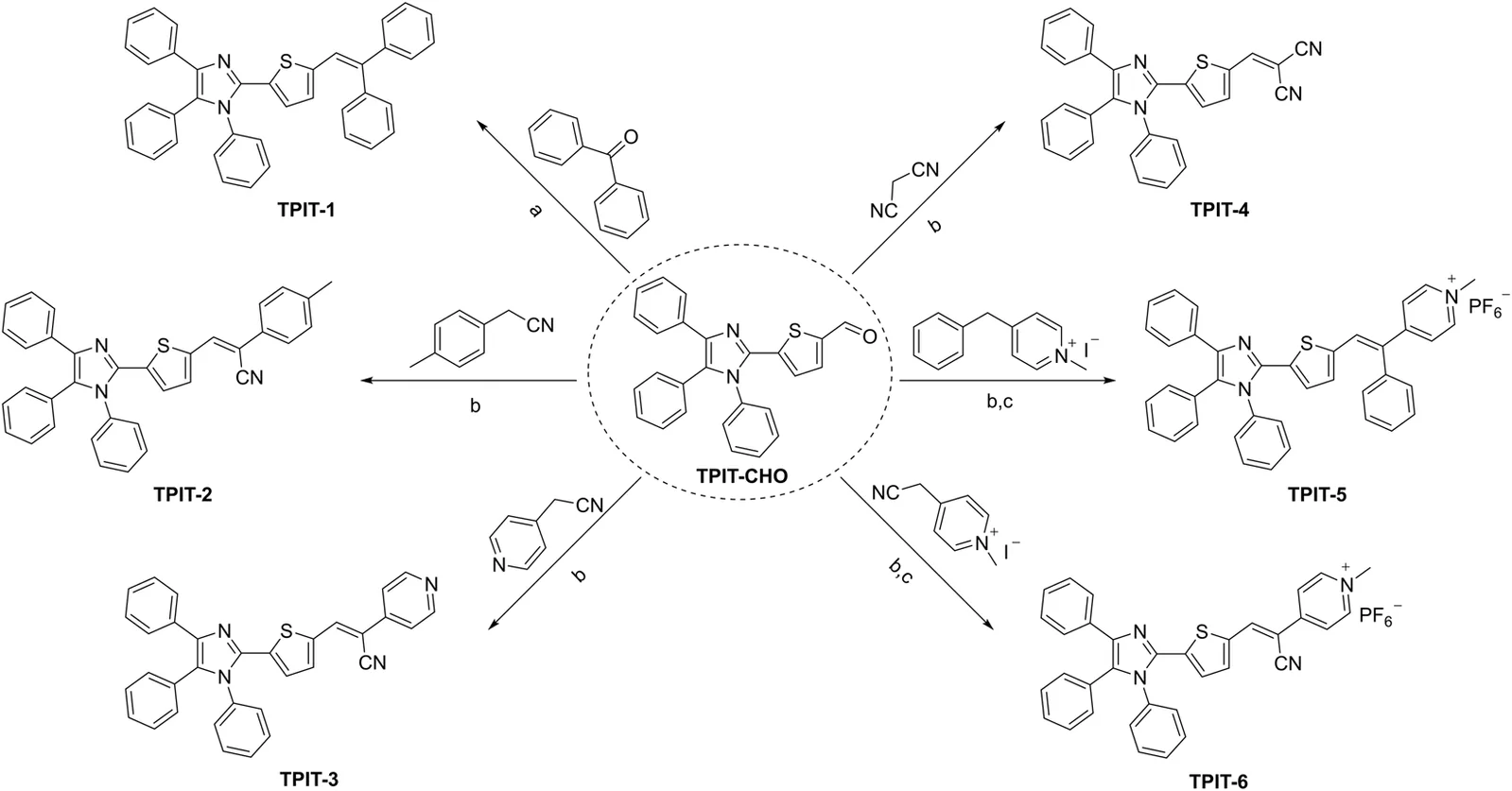
Synthetic routes to TPIT-X molecules. Reagents and conditions: **(a)** TiCl_4_, Zn, THF, reflux, 10–12 h; **(b)** piperidine, EtOH, reflux, 10–12 h; **(c)** KPF_6_, acetone, room temperature.

Constructing molecular structures with an electron donor–acceptor (D–A) backbone is widely recognized as an effective approach to enhance the internal charge transfer (ICT) effect of fluorescent molecules and thereby extending their emission wavelengths (Ersoy and Henary, 2025; Essam et al., 2021; Jaswal and Kumar, 2020; İçli et al., 2010). The rational selection and systematic integration of D-A units are critical for successful design of D-A fluorophores with high luminescence yield and red-shifted emission wavelength. Imidazole, an electron-rich heterocyclic structure containing two nitrogen atoms, has been receiving increasing attention in different fields such as bio-labeling, fluorescent sensors, organic light emitting diodes (OLEDs) and light sources due to its favorable biological activity and photoelectric properties (Tolomeu and Fraga, 2023; Verma et al., 2013). Notably, it has a unique bipolar configuration of two donor and acceptor nitrogen atoms apart via C2. Introducing electron-withdrawing or neutral substituents at the C2 position enhances the electron-donating behavior of the nitrogen-containing heterocyclic system. Conversely, functionalizing the 2-position of the imidazole scaffold with an electron-donating moiety induces electron-withdrawing properties, thereby reversing the electronic polarization of the heterocycle (Al Sharif et al., 2023). Given these unique electronic characteristics and facile modification on the five-membered ring, imidazole derivatives also serve as ideal candidates for electron donors in D–A type fluorophores. Among numerous imidazole derivatives, triphenylimidazole has caught our attention due to its characteristic property: the intramolecular rotations of multiple benzene rotors on the imidazole ring are supposed to induce nonradiative decay, thereby quenching the fluorescence of the molecule in dilute solutions. In contrast, within the aggregated state, steric hindrance from molecular packing significantly restricts these rotations, leading to restored fluorescence intensity. This restriction of intramolecular rotation (RIR) effect is widely recognized as the primary contributor to the aggregation-induced emission (AIE) phenomenon (Leung et al., 2014). Built in nonplanar 1,4,5-triphenylimidazole core structure, we conceived a kind of AIE molecular platform, TPIT-X, with a classic D-A framework and further extended its conjugation length by incorporating thiophene as a π-bridge, forming a “D-π-A” type system. By conjugating a series of acceptor units with different electron affinity and steric hindrance, the emission wavelengths and AIE properties of TPIT-X could be tuned as desired. Based on this engineering strategy, TPIT-1 to TPIT-6 were conceived in this study. The triphenylimidazole moiety, functioning as the core structural unit in this molecular series, exhibits a bipolar electronic characteristic that enables its reversible switching between electron-donating and electron-withdrawing roles. In the TPIT-1 molecule, this heterocyclic system acts as a weak electron-withdrawing unit due to π-conjugation with electron-donating diphenylethylene, which delocalizes electron density. However, the introduction of electron-withdrawing groups such as cyanide, pyridyl, and pyridinium onto the conjugated vinyl group of the imidazole transforms the tricyclic core into an electron donor. All these molecules were synthesized via facile routes. Specifically, we synthesized the core donor structure TPIT-CHO according to previous reports, from which all other target products were obtained from a single-step reaction (Scheme 1). TPIT-1 was prepared by McMurry coupling reaction between TPIT-CHO and benzophenone. TPIT-2, TPIT-3, TPIT-4, TPIT-5, TPIT-6 were obtained through condensation reactions between TPIT-CHO and 2-phenylacetonitrile, 2-(pyridin-4-yl)acetonitrile, malononitrile, 4-benzyl-1-methyl-pyridinium iodide, 1-methyl-4-(prop-2-yn-1-yl)pyridin-1-ium iodide, respectively. The iodide anions (I^−^) in TPIT-5 and TPIT-6 were further replaced with hexafluorophosphate ions (PF_6_^−^) using potassium hexafluorophosphate (KPF_6_) in acetone. All these structures were confirmed by proton nuclear magnetic resonance (H-NMR) spectroscopy and high-resolution mass spectrometry (HRMS) (Supplementary Figurea S1–S14).

### Optical properties

**FIGURE 1:**
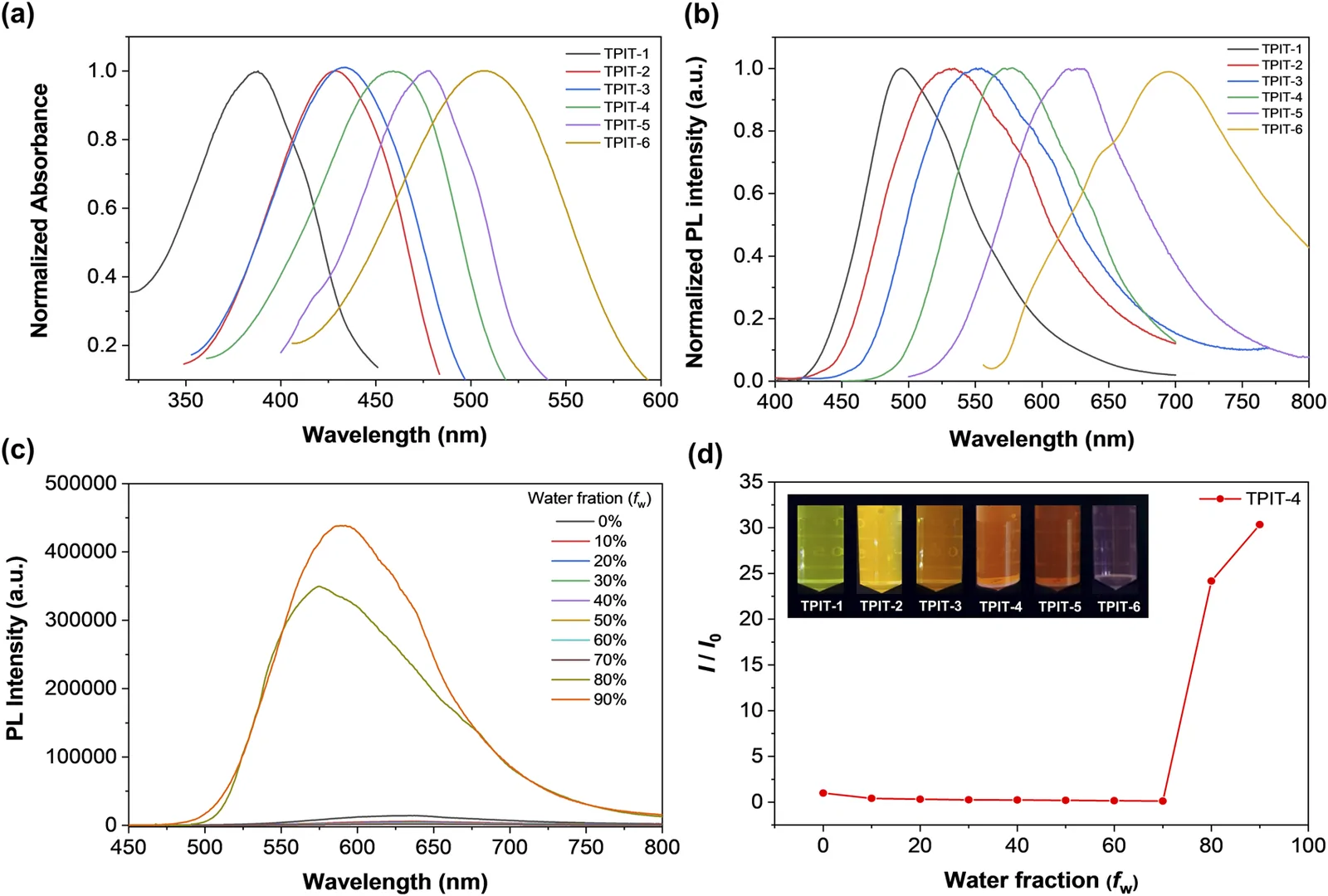
**(a)** Normalized UV-Vis absorption spectra of TPIT-1 to TPIT-6 in tetrahydrofuran (THF). **(b)** Normalized fluorescence emission spectra of TPIT-1 to TPIT-6 in THF. **(c)** Fluorescence emission spectra of TPIT-4 in water/THF mixtures with varying water fractions (*f*_w_). **(d)** Plot of PL peak intensities of TPIT-4 as a function of *f*_w_ in water/THF mixtures. *I*_*0*_ and *I* denote the PL intensities of the compounds in pure THF and in water/THF mixtures, respectively. Inset: Fluorescent photographs of TPIT-1 to TPIT-6 in THF/water mixtures (*f*_w_ = 90%) under 365 nm excitation.

The optical properties of TPIT-1 to TPIT-6 were firstly investigated by UV-vis absorption and photoluminescence (PL) spectroscopy. As illustrated in Figures 1a,b, the absorption maxima of TPIT-1 to TPIT-6 progressively red-shifted from 388 nm to 507 nm, while their emission maxima spanned from 490 nm (green) to 695 nm (deep red). These results demonstrate successful tuning of emission wavelengths across almost entire visible spectrum by solely modifying the acceptor moieties within the D-π-A backbone. The redshift in both absorption and emission can be attributed to the gradually increasing electron-withdrawing capability of the acceptor units (phenyl < cyano < pyridyl), which effectively strengthens the ICT effect. The AIE characteristics of these fluorescent probes were also investigated in tetrahydrofuran (THF)/water mixed solvents at different water contents (*f*_w_). As a typical example showed in Figures 1c,d, TPIT-4 consistently exhibited negligible fluorescence intensity when the water fraction (*f*_w_) increased from 0% to 70%, whereas a marked 30.3-fold enhancement emerged when *f*_w_ exceeded 90%. Similar trends were observed for other derivatives (Supplementary Figures S15-S19), together with the photographs of their powders under 365 nm excitation (Supplementary Figure S20), confirming the typical AIE characteristics of these molecules. This phenomenon can be attributed to the RIR mechanism, where the restriction of intramolecular rotation in aggregated state suppresses non-radiative decay pathways. Notably, The fluorescence quenching of TPIT-5 and TPIT-6 at low water content (*f*_w_ < 60%) is primarily attributed to efficient electron transfer from the imidazole donor to the N-methylpyridinium acceptor, which induces substantial charge separation and promotes the formation of a twisted intramolecular charge-transfer (TICT) state. Under these conditions, increased solvent polarity facilitates a nearly orthogonal donor–acceptor conformation through excited-state bond rotation, ultimately leading to emission attenuation (McDonald et al., 2025; Wang et al., 2021). In contrast, when the water fraction exceeds 60%, molecular aggregation effectively restricts internal bond rotation, thereby suppressing the TICT pathway and, consistent with the RIR mechanism, triggering a pronounced AIE response (Bhuin et al., 2025; Zhang et al., 2024; Zhu et al., 2025). In conclusion, these results prove that the emission window and AIE performance of this molecular “platform” can be precisely programmed through rational selection of acceptor units with distinct electron-withdrawing abilities and steric effects.

### Cell imaging

**FIGURE 2:**
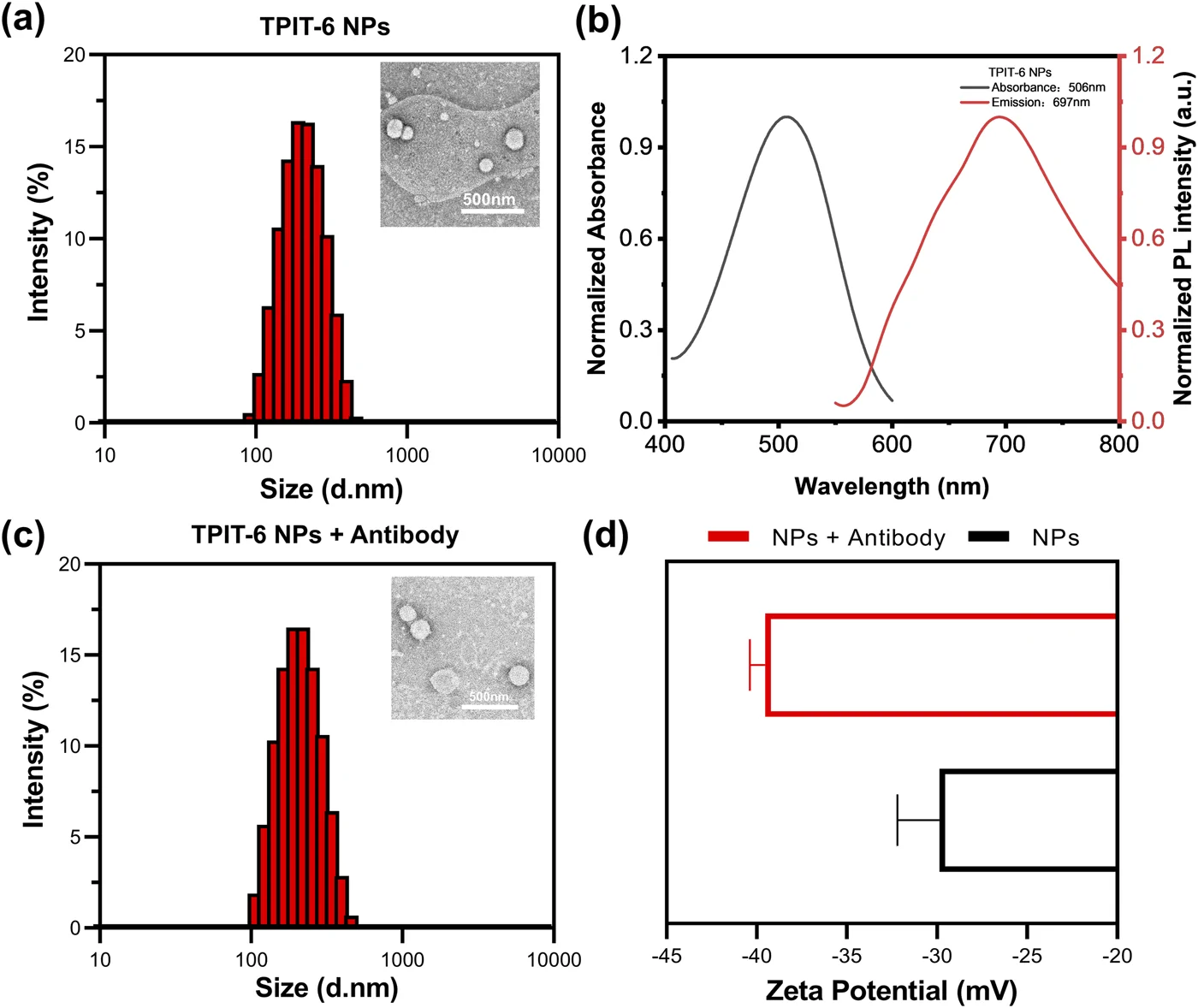
**(a)** Dynamic light scattering (DLS) diameter of TPIT-6 NPs; Inset: Transmission electron microscopy (TEM) image of the NPs. **(b)** UV-Vis absorption and photoluminescence spectra of TPIT-6 NPs. **(c)** Dynamic light scattering (DLS) diameter of TPIT-6 NPs-Ab; Inset: Transmission electron microscopy (TEM) image of the NPs. **(d)** Comparison of the zeta potential between TPIT-6 NPs and TPIT-6 NPs-Ab. Error bars: standard deviation (SD), experimental times: n = 3.

It is well known that long-wavelength emitting fluorophores can effectively avoid biological autofluorescence, reduce photon scattering, and improve deep-tissue penetration, thereby enhancing imaging contrast and resolution. Therefore, we selected TPIT-6, which emits in deep red and NIR-I region, as the fluorescent probe for *in vivo* imaging. To overcome the poor solubility of TPIT-6 in aqueous media, we prepared a suspension of TPIT-6 nanoparticles (TPIT-6 NPs) at a concentration of 2 mg/mL using DSPE-PEG2000 lipids. As illustrated in Figure 2a, dynamic light scattering (DLS) and transmission electron microscopy (TEM) characterization confirm that TPIT-6 NPs exhibit a uniform, nearly spherical morphology with an average diameter of approximately 200 nm. Further fluorescence spectroscopy analysis (Figure 2b) reveals that TPIT-6 NPs have a maximum absorption wavelength at 506 nm, with an emission peak located at 697 nm. These results demonstrate the successful establishment of the TPIT-6 NPs deep-red-emitting probe system.

**FIGURE 3:**
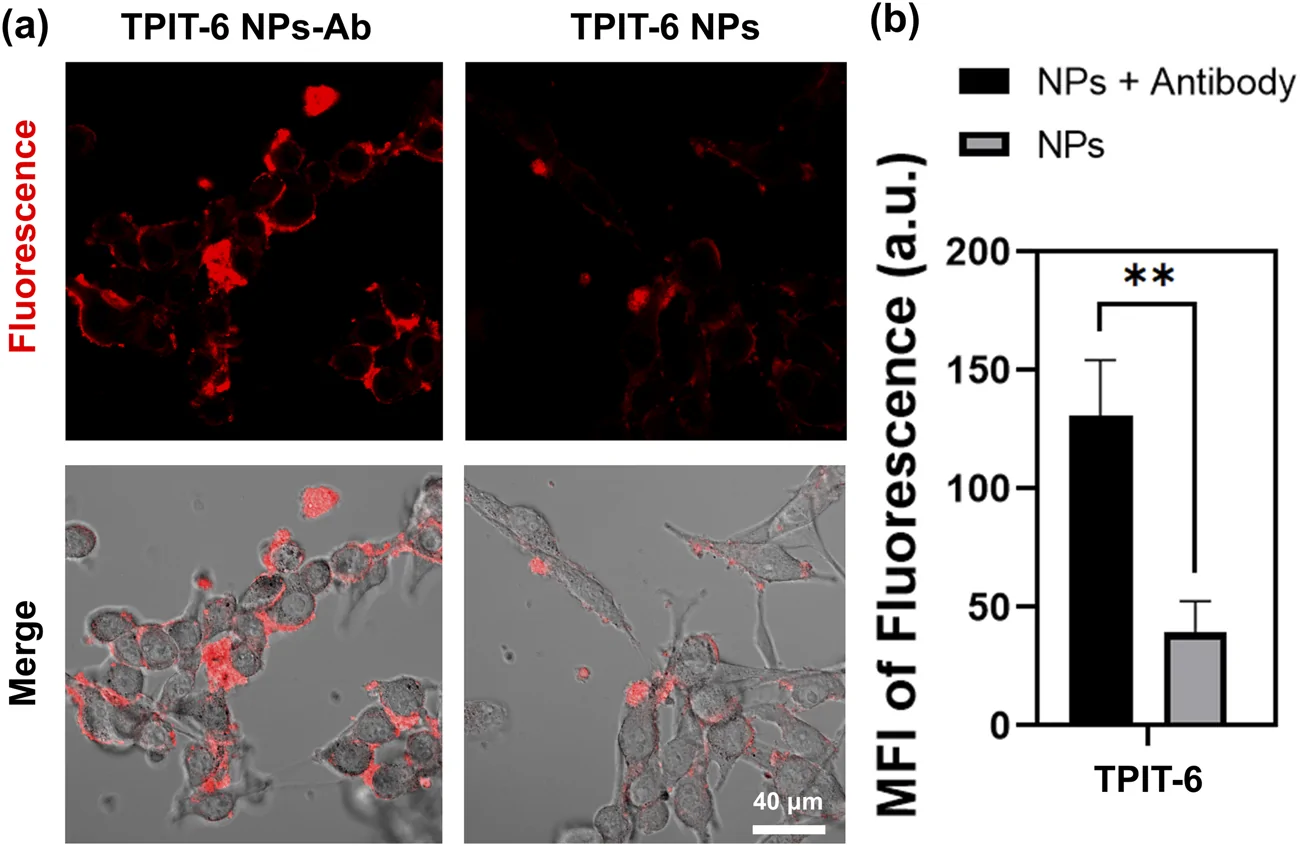
**(a)** Confocal fluorescence images of SCC7 cells treated with (A) TPIT-6-NPs (10 μM) and **(b)** TPIT-6-NPs-Ab (10 μM). Red color indicates the fluorescence signal in the red channel (680–720 nm, λ_ex_ = 506 nm). Merge: Merged images with red channel and bright-field images. B) Mean fluorescence intensity of TPIT-6-NPs and TPIT-6-NPs-Ab. Error bars: standard deviation (SD), experimental times: n = 3, **P < 0.01. Scale bar: 40 μm.

To equip the NPs with active targeting capability towards SCC tumor, we functionalized TPIT-6 NPs by coupling anti-EGFR antibodies, resulting in TPIT-6 NPs-Ab. Characterization by DLS and TEM confirmed that the resulting TPIT-6 NPs-Ab also exhibited a uniform near-spherical morphology with an average diameter of approximately 200 nm (Figure 2c). As shown in Figure 2d, the zeta potentials of TPIT-6 NPs-Ab and TPIT-6 NPs were measured to be −39 mV and −29 mV, respectively. According to previous reports, a higher absolute zeta potential value typically indicates better suspension stability, as enhanced electrostatic repulsion between particles reduces their tendency to aggregate (Jarzynska et al., 2025). This finding suggests that antibody conjugation moderately improves the stability of the nanoparticles. In addition, neither TPIT-6 NPs nor TPIT-6 NPs-Ab exhibited any significant variation in fluorescence intensity following a 10-day storage period in PBS buffer at 37 °C, indicating excellent stability under physiological conditions (Supplementary Figure S21). Before initiating cell experiments, the cytotoxicity of TPIT-6 NPs and TPIT-6 NPs-Ab toward normal cells was evaluated in mouse smooth muscle cells (SMCs) using the 3-(4,5-dimethylthiazol-2-yl)-2,5-diphenyltetrazolium bromide (MTT) assay. As shown in Supplementary Figures S22 and S23, after 24 h of co-incubation with TPIT-6 NPs and TPIT-6 NPs-Ab under dark, respectively, the viability of SMCs remained above 90% in both cases even when the probe concentration was increased to 100 μM. These findings indicate the low cytotoxicity of TPIT-6 NPs and TPIT-6 NPs-Ab toward normal cells and tissues. To further assess the effectiveness of anti-EGFR antibodies in SCC targeting, SCC7 cells were incubated with either TPIT-6 NPs or TPIT-6 NPs-Ab. As shown in Figure 3, confocal laser scanning microscopy demonstrated that TPIT-6 NPs-Ab specifically bound to SCC7 cells, while TPIT-6 NPs showed minimal binding under identical conditions. It is noteworthy that both NPs appear to predominantly localize on the cell surface or within intercellular spaces, demonstrating that nanospheres of this size exhibit limited cellular internalization efficiency.

### *Ex vivo* tissue imaging

**SCHEME 2:**
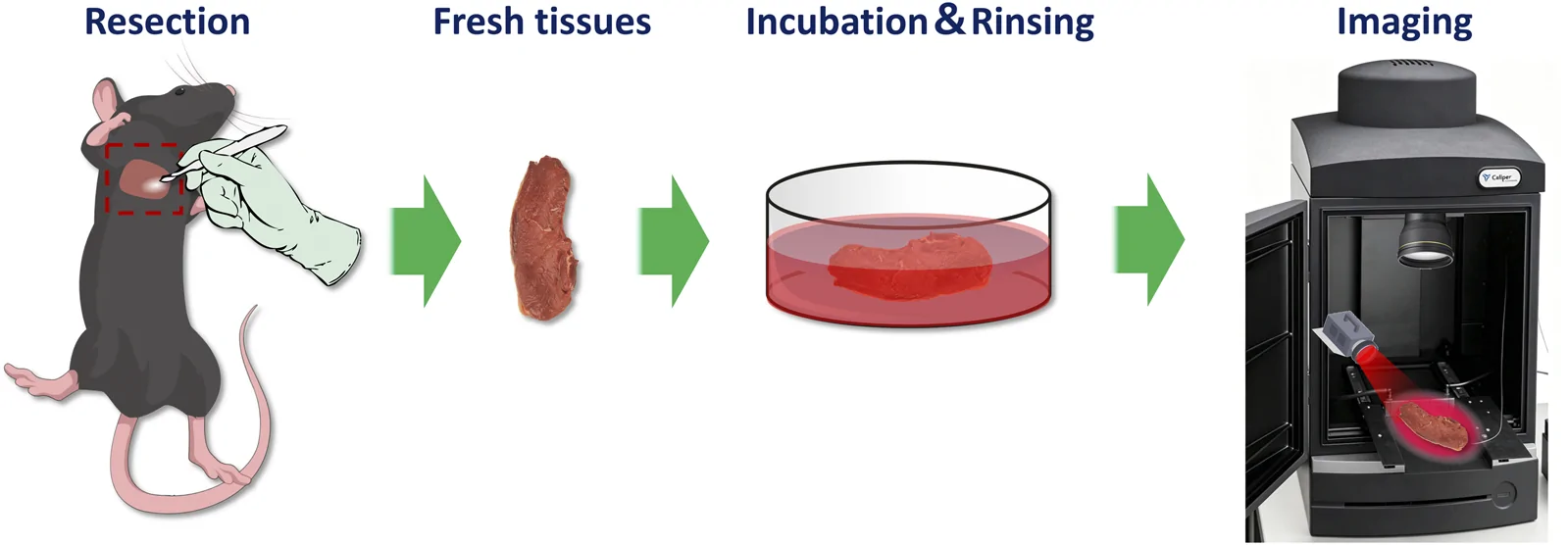
Experimental procedure for suspected tissue examination. Tissue specimens from the surgical margin were incubated with TPIT-6-NPs-Ab (50 μM) at room temperature for 15 min, washed twice with PBS, and then imaged using an IVIS LumiNova II system.

**FIGURE 4:**
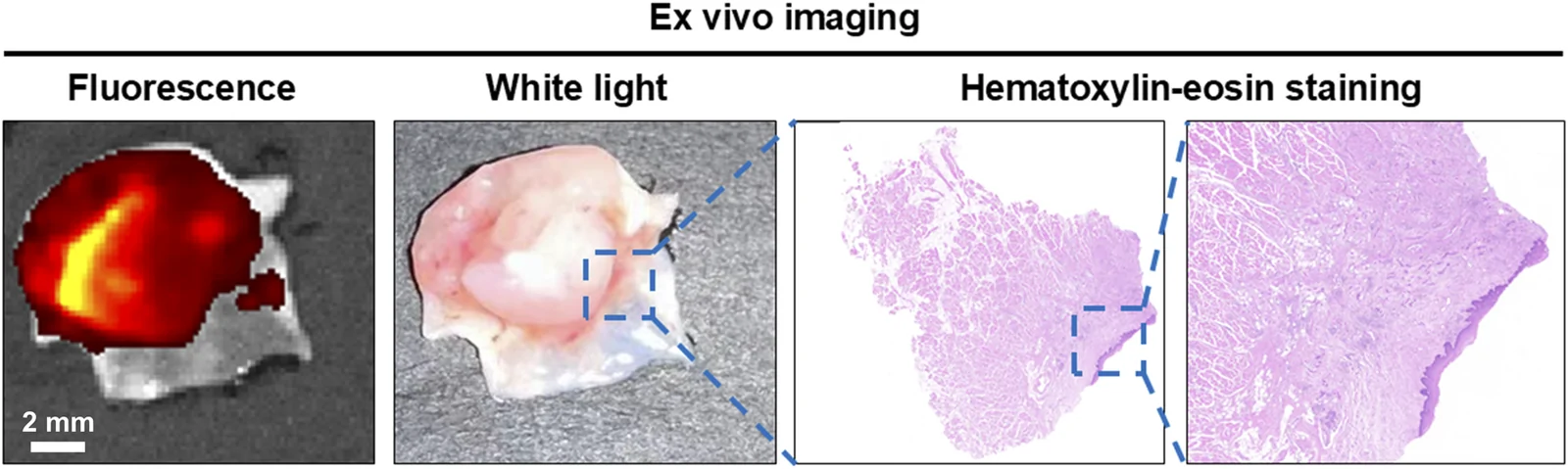
Fluorescence and white-light images of an SCC7 tumor tissue slice following *ex vivo* incubation with TPIT-6-NPs-Ab, along with hematoxylin and eosin (H&E) staining of the paraffin section for pathological evaluation. Imaging channel: 695–770 nm. λ_ex_ = 500 nm. Scale bar: 2 mm.

We next explored whether the TPIT-6 NPs-Ab probe could serve as a rapid diagnostic tool for intraoperative tissue biopsy. As shown in Scheme 2, we designed a convenient testing workflow to obtain diagnostic information including tumor margin discrimination and tumor cell metastasis status during the surgical procedure, thereby assisting surgeons in rapid pathological diagnosis. In detail, suspected tumor specimens resected from the surgical margin were immediately placed in PBS containing 5% fetal bovine serum, then transferred to PBS containing 50 μM TPIT-6 NPs-Ab and incubated at room temperature for 15 min. Following incubation, the specimen was rinsed twice with PBS buffer to remove unbound probes and then imaged using an IVIS LumiNova II imaging system. *Ex vivo* experiments were then conducted in C3H mice models to evaluate the performance of our method in the pathological diagnosis of SCC subcutaneous tumor resection. As shown in Figure 4, after treatment with TPIT-6 NPs-Ab, certain areas of the suspected tumor tissue specimens became highly luminescent and the margins of these areas are quite clear under imaging system. To validate the accuracy of the tumor margin indicated by fluorescence, tissue samples were also collected from the edges of the fluorescent regions and processed into paraffin sections. Subsequent hematoxylin and eosin (H&E) staining of these sections revealed the coexistence of both tumor cells and normal tissues under microscopic examination. Accordingly, the fluorescent regions reasonably corresponded to the actual tumor locations. These findings indicate that TPIT-6 NPs-Ab-based fluorometric assay can discriminate tumor margins rapidly through *ex vivo* specimens lighted by fluorescence during resection surgeries.

## Conclusion

In this study, we report an effective *ex vivo* biopsy method for hand SCC tumor resection. Central to this method is a novel family of AIEgens, which was developed from multi-rotor imidazole derivatives, enjoying facile synthesis and wide color tunability. By incorporating various acceptor units into the D-π-A backbone, the emission window can be tuned from green (490 nm) to deep red (695 nm), almost covering the entire visible light spectrum. Optical evaluation demonstrated their typical AIE characteristics (up to 30-fold brightness enhancement). For biomedical applications, TPIT-6 was encapsulated within DSPE-PEG2000 lipids to prepared NPs, which were further coupled with anti-EGFR antibodies. After confirming the precise targeting capability of TPIT-6 NPs-Ab in cell experiments, we designed an intraoperative pathological diagnosis method based on this probe and evaluated it during SCC subcutaneous tumor resection in C3H mice. Fluorescence imaging of freshly excised marginal tissues revealed that this approach effectively allowed rapid identification of tumor boundaries within the tissue specimens. This work not only provide an innovative strategy for developing wide-emission-range AIE-active fluorophores but also broaden their potential application in intraoperative tumor biopsy.

## Experimental

### Materials

Aniline was obtained from Lingfeng Chemical Reagent Co., Ltd. (Shanghai, China). Benzil, 2,5-Thiophenedicarboxaldehyde, ammonium acetate, Benzophenone, 2-(Pyridin-4-yl)acetonitrilewere, 4-Methylbenzyl cyanide were purchased from Bide Pharmatech Co., Ltd. (Shanghai, China). Malononitrile, 4-Benzylpyridine were purchased from Yien Chemical Technology Co., Ltd. (Shanghai, China). Zinc powder was purchased from Sigma-Aldrich Trading Co., Ltd. (Shanghai, China). Acetic acid, THF, ethanol (EtOH) and TiCl_4_ were purchased from J&K Scientific Ltd. (Beijing, China). All other chemicals and solvents of high purity were purchased and used without further purification. All reactions dealing with air-sensitive compounds were performed under a nitrogen atmosphere.

### Measurement

UV-Vis absorption spectra were measured using a Shimadzu UV-1900i spectrophotometer in the spectrofluorometer, with the excitation wavelength fixed at the absorption maximum of each sample and emission collected between 400 and 800 nm. ^1^H NMR spectra were obtained on a Bruker Avance 400 MHz spectrometer. High-resolution mass spectrometry (HRMS) data were acquired using an Agilent LC-QTOF-MS system. Cell imaging was conducted on a Zeiss LSM710 confocal microscope, and murine tissue imaging was performed on an IVIS LumiNova II system.

#### Synthesis of compound TPIT-CHO

A solution of aniline (558 mg, 6.0 mmol) and 2,5-thiophenedicarboxaldehyde (840 mg, 6.0 mmol) in acetic acid (50 mL) was stirred at room temperature for 1 h. Subsequently, benzil (1.26 g, 6.0 mmol) and ammonium acetate (3.23 g, 6.0 mmol) were added, and the reaction mixture was heated at 120 °C for 8 h. Upon completion, the solvent was removed under reduced pressure. The residue was extracted with dichloromethane (3 × 70 mL), and the combined organic layers were concentrated. The crude product was purified by column chromatography on silica gel using DCM as the eluent to afford 1.33 g of a yellow solid in 54.8% yield. ^1^H NMR (400 MHz, CDCl_3_) δ 9.80 (s, 1H), δ 7.58 (s, 1H), δ 7.51 (s, 1H), δ 7.47–7.38 (m, 3H), δ 7.28–7.19 (m, 9H), δ 7.17–7.14 (m, 2H), δ 6.98 (s, 1H). ^13^C NMR (101 MHz, CDC13) δ 182.86, 142.95, 142.10, 140.95, 139.32, 136.24, 135.90, 133.62, 132.64, 130.92, 129.93, 129.76, 129.68, 128.89, 128.51, 128.47, 128.31, 127.33, 127.14, 126.77, 77.38, 77.27, 77.07, 76.75. HRMS, *m/z* calcd. for C_26_H_18_N_2_OS: 406.1140; found 406.5030 (M + H)^+^.

#### Synthesis of compound TPIT-1

A mixture of TPIT-CHO (0.5 mmol, 203 mg), benzophenone (1.5 mmol, 273.3 mg), and zinc dust (4.8 mmol, 313.7 mg) in dry THF (15 mL) was stirred at −40 °C for 10 min. Subsequently, TiCl_4_ (2.4 mmol, 0.263 mL) was added dropwise slowly. The reaction mixture was then heated under reflux for 10–12 h. Upon completion, the mixture was cooled to room temperature and filtered under vacuum. The filtrate was extracted with dichloromethane (3 × 30 mL), and the combined organic layers were concentrated under reduced pressure. The crude product was purified by column chromatography on silica gel using dichloromethane as the eluent to afford 111 mg of a yellow solid in 40% yield. ^1^H NMR (400 MHz, CDCl_3_) δ 7.55 (s, 1H), δ 7.53 (s, 1H), δ 7.41–7.39 (m, 3H), δ 7.36–7.33 (m, 2H), δ 7.30–7.06 (m, 20H), δ 6.62 (d, J = 3.8 Hz, 1H). ^13^C NMR (101 MHz, CDCl3) δ 139.09, 130.98, 130.05, 129 34, 128.89, 128.29, 128.20, 128.15, 127.47,127.37, 126.87, 120.55, 77.36, 77.24, 77.04, 76.72, 39.30. HRMS, *m/z* calcd. for C_39_H_28_N_2_S: 556.1973; found 557.2051 (M + H)^+^.

#### Synthesis of compound TPIT-2

To a solution of TPIT-CHO (0.4 mmol, 162.4 mg) and 4-methylacetonitrile (0.44 mmol, 57.6 mg) in dry EtOH, a solution of NaOH (50 mg) in EtOH was added dropwise at room temperature. The reaction mixture was further stirred for 4 h. Upon completion of the reaction. The crude product was purified by column chromatography on silica gel using dichloromethane as the eluent to afford 147.5 mg of a yellow solid in ​​73% yield. ^1^H NMR (400 MHz, CDCl_3_) δ 7.62–7.60 (m, 4H), δ 7.33–7.06 (m, 17H), 6.78 (d, J = 4.2 Hz, 1H), 2.37 (s, 3H). ^13^C NMR (101 MHz, CDCl3) δ 148.5, 142.3, 138.7, 138.2, 137.5, 135.7, 134.3, 133.0, 131.7, 130.1, 129.6, 129.2, 128.7, 128.6, 127.9, 127.5, 126.4, 122.1, 118.8, 105.5. HRMS, *m/z* calcd. for C_35_H_25_N_3_S: 519.1769; found 520.1846 (M + H)^+^.

#### Synthesis of compound TPIT-3

A solution of TPIT-CHO (0.4 mmol, 162.4 mg) and malononitrile (0.44 mmol, 29 mg) in dry EtOH was refluxed for 10 h in the presence of one or two drops of piperidine as catalyst. Upon completion of the reaction. The crude product was purified by column chromatography on silica gel using dichloromethane as the eluent to afford 130 mg of a yellow solid in ​​72% yield​​. ^1^H NMR (400 MHz, CDCl_3_) δ 8.63 (s, 2H), δ 7.67 (s, 1H), δ 7.61 (d, J = 7.5 Hz, 2H), δ 7.55 (m, 1H), δ 7.48–7.39 (m, 5H), δ 7.33–7.11 (m, 10H),6.81 (d, J = 4.0 Hz, 1H). ^13^C NMR (101 MHz, CDCI3) δ 267.05, 151.00, 150.44, 141.49, 141.02, 139.37, 139.28, 136.94, 136.18, 136.03, 135.32, 134.03, 133.71, 132.49, 130.93, 130.84, 129.90, 129.75, 129.56, 128.96, 128.82, 128.50, 128.44, 128.29, 127.34, 127.26, 127.09, 126.74, 119.53, 117.06, 105.11, 77.40, 77.28, 77.09, 76.77. HRMS, *m/z* calcd. for C_33_H_22_N_4_S: 506.1565; found 507.1641 (M + H)^+^.

#### Synthesis of compound TPIT-4

A solution of TPIT-CHO (0.4 mmol, 162.4 mg) and 4-pyridineacetonitrile (0.44 mmol, 51.9 mg) in dry EtOH was refluxed for 10 h in the presence of one or two drops of piperidine as catalyst. Upon completion of the reaction. The crude product was purified by column chromatography on silica gel using dichloromethane as the eluent to afford 178.1 mg of a yellow solid in ​​88% yield​​. ^1^H NMR (400 MHz, CDCl_3_) δ 7.54 (s, 1H), δ 7.52 (m, 3H), 7.50 (m, 4H), δ 7.48 (m, 8H), 7.46 (d, J = 7.5 Hz, 2H). ^13^C NMR (101 MHz, CDCl_3_) δ 182.84, 149.95, 137.98, 135.51, 134.83, 130.82, 130.38, 129.98, 128.85, 128.70, 128.58, 128.35, 127.42, 127.39, 115.43, 114.19, 113.14, 77.34, 77.23, 77.02, 76.71, 29.71, 0.01. HRMS, *m/z* calcd. for C_29_H_18_N_4_S: 454.1252; found 455.1330 (M + H)^+^.

#### Synthesis of compound TPIT-5

4-Benzyl-1-methylpyridium iodide was first prepared through methylation of 4-benzylpyridine. A solution of TPIT-CHO(0.4 mmol, 162.4 mg) and 4-benzyl-1-methylpyridium iodide (0.4 mmol, 124.4 mg) in dry EtOH was refluxed for 12 h in the presence of one or two drops of piperidine as catalyst. Upon completion of the reaction. The crude product was purified by column chromatography on silica gel using dichloromethane as the eluent to afford 130 mg of a yellow solid in ​​72% yield​​. ^1^H NMR (400 MHz, CDCl_3_) δ 8.82 (d, J = 6.3 Hz, 2H), δ 8.37 (d, J = 6.3 Hz, 2H), δ 8.02 (s, 1H), δ 7.58 (m, 3H), δ 7.44 (m, 4H), 7.25 (m, 8H), δ 7.88 (m, 7H), 7.13 (d, J = 7.2 Hz, 2H), δ 6.52 (s, 1H), 4.47 (s, 3H). ^13^C NMR (101 MHz, CDCl_3_) δ 157.0, 148.5, 146.3, 142.3, 140.0, 138.7, 138.2, 137.5, 133.0, 131.7, 130.1, 129.6, 129.2, 128.7, 128.6, 128.3, 128.2, 127.9, 127.5, 124.8, 122.1, 49.0 HRMS, *m/z* calcd. for C_39_H_30_N_3_S^+^: 572.2155; found 522.2157 M^+^.

#### Synthesis of compound TPIT-6

4-(cyanomethyl)-1-methylpyridin-1-ium iodide was first prepared through methylation of 2-(pyridin-4-yl)acetonitrile. A solution of TPIT-CHO (0.4 mmol, 162.4 mg) and 4-(cyanomethyl)-1-methylpyridin-1-ium iodide (0.4 mmol, 124 mg) in dry EtOH was refluxed for 12 h in the presence of one or two drops of piperidine as catalyst. Upon completion of the reaction. The crude product was purified by column chromatography on silica gel using dichloromethane as the eluent to afford 165.9 mg of a yellow solid in ​​64% yield. ^1^H NMR (400 MHz, CDCl_3_) δ 8.78 (d, J = 6.5 Hz, 2H), δ 8.29 (s, 1H), δ 7.83 (d, J = 6.5 Hz, 2H), δ 7.63 (m, 3H), δ 7.48–7.36 (m, 6H), δ 7.27 (m, 7H), δ 6.53 (s, 1H), 4.24 (s, 3H). ^13^C NMR (101 MHz, CDCl_3_) δ 157.0, 148.5, 146.3, 142.3, 138.7, 138.2, 137.5, 133.0, 131.7, 130.1, 129.6, 129.2, 128.7, 128.2, 127.5, 124.8, 122.1, 49.0. HRMS, *m/z* calcd. for C_34_H_25_N_4_S^+^: 521.1794; found 521.1799 M^+^.

### Preparation of nanoparticles

Lipophilic TPIT-6 (1 mg) and the amphiphilic polymer DSPE-PEG2000 (2 mg; 1,2-distearoyl-sn-glycero-3-phosphoethanolamine-N-[methoxy (polyethylene glycol)-2000]) were dissolved together in 1.0 mL of tetrahydrofuran (THF). The resulting solution was then poured into 10 mL of ultrapure water under continuous ultrasonication for 10 min. Afterward, the residual THF was eliminated by flushing with nitrogen gas, and the obtained nanoparticle suspension was kept at 4 °C until further use. The concentration of the resulting nanoparticles (NPs) was determined to be 120 μmol·mL^-1^.

### Cellular imaging

SCC7 mouse squamous cell carcinoma cells were obtained from the American Type Culture Collection (ATCC). Cell cultures were maintained at 37 °C in a humidified, 5% CO_2_ atmosphere using DMEM supplemented with 10% fetal bovine serum (FBS) and 1% penicillin-streptomycin. For confocal imaging, SCC7 cells were plated into confocal dishes at a density of 2 × 10^5^ cells/mL in RPMI-1640 medium and left to adhere overnight. After the medium was removed and the cells were washed three times with PBS, they were incubated with RPMI-1640 containing 10 μM TPIT-6 NPs or TPIT-6 NPs-Ab for 4 h. Following a further three PBS washes, the cells were resuspended in 1 mL of PBS and imaged using a confocal microscope (Zeiss LSM710) with 488 nm excitation and appropriate emission filters.

### Cytotoxicity study

Smooth muscle cells (SMCs) in the logarithmic growth phase were maintained at 37 °C in a 5% CO_2_ atmosphere until they reached confluence in 96-well plates. Ten wells were randomly chosen and assigned to two groups, which were then treated with varying concentrations of TPIT-6 NPs (10–50 mmol·L^-1^, based on the chromophore standard) for 24 h under either light or dark conditions. Thereafter, 20 μL of MTT reagent (3-(4,5-dimethylthiazol-2-yl)-2,5-diphenyltetrazolium bromide, 5 mg·mL^-1^) was added to each well, and the plates were incubated for an additional 4 h. The culture medium was then removed, and 100 μL of DMSO was added to dissolve the formazan crystals. The plates were gently agitated on an orbital shaker for 10 min, and the absorbance at 550 nm was recorded using a microplate reader. The obtained data were plotted as bar graphs.

### *Ex vivo* imaging

All animal experiments were approved by the ethics committee and conducted in accordance with relevant guidelines. Female C3H mice weighing 18–22 g were obtained from Beijing VITON-LH Experimental Technology Co., Ltd. The animals were maintained under a 12-h light/12-h dark cycle at 25 °C and 50%–60% relative humidity, with *ad libitum* access to drinking water and standard diet. The health status of the mice was checked daily, and all experiments commenced following a one-week acclimatization period. Subsequently, female C3H mice were used to establish SCC7 tumors by intraperitoneal administration of 100 μL of a cell suspension containing 3 × 10^6^ SCC7 cells. Tumor resection was carried out when the tumors grew to approximately 10 mm. After tumor removal, tissue specimens suspected of containing tumor cells were harvested from the surgical margin and promptly immersed in PBS supplemented with 5% fetal bovine serum. Subsequently, they were transferred to PBS containing 50 μM TPIT-6 NPs-Ab and kept at ambient temperature for 15 min. After the incubation, the specimens were each washed twice with PBS to eliminate unbound probes, followed by imaging with an IVIS LumiNova II imaging system.

## Data Availability

The original contributions presented in the study are publicly available. This data can be found here: https://doi.org/10.6084/m9.figshare.33159095. All other experimental data presented in the study are included in the article/Supplementary Material.
