# Supplementary material for: Multi-rotor imidazole-based fluorophores for rapid delineation of squamous cell carcinoma infiltration margins in murine surgical specimens
Source: Front Chem. 2026 Aug 10;14:1913502. doi: 10.3389/fchem.2026.1913502 (PMC13500108; doi:10.3389/fchem.2026.1913502)
Supplement: Supplementary file 1 [file DataSheet1.docx]

*Supporting information for*

**Multi-Rotor Imidazole-Based Fluorophores for Rapid Delineation of Squamous Cell Carcinoma Infiltration Margins in Murine Surgical Specimens**

Huabin He^1^*

1. *Department of Hand and Foot Surgery, Yiwu Central Hospital, 699 Jiangdong Road, Yiwu, Zhejiang Province, PR China.* **Email:* *hehuabin2@163.com*

Fig. S1 LC-QTOF-MS spectrum of **TPIT-CHO**.

Fig. S2 ^1^H NMR spectrum of **TPIT-CHO**.

Fig. S3 LC-QTOF-MS spectrum of **TPIT-1**.

Fig. S4 ^1^H NMR spectrum of **TPIT-1**.

Fig. S5 LC-QTOF-MS spectrum of **TPIT-2**.

Fig. S6 ^1^H NMR spectrum of **TPIT-2**.

Fig. S7 LC-QTOF-MS spectrum of **TPIT-3**.

Fig. S8 ^1^H NMR spectrum of **TPIT-3**.

Fig. S9 LC-QTOF-MS spectrum of **TPIT-4**.

Fig. S10 ^1^H NMR spectrum of **TPIT-4**.

Fig. S11 LC-QTOF-MS spectrum of **TPIT-5**.

Fig. S12 ^1^H NMR spectrum of **TPIT-5**.

Fig. S13 LC-QTOF-MS spectrum of **TPIT-6**.

Fig. S14 ^1^H NMR spectrum of **TPIT-6**.

Fig. S15. Fluorescence emission spectra of **TPIT-1** versus water fraction (*f_w_*) in water/tetrahydrofuran (THF) mixtures.

Fig. S16. Fluorescence emission spectra of **TPIT-2** versus water fraction (*f_w_*) in water/tetrahydrofuran (THF) mixtures.

Fig. S17. Fluorescence emission spectra of **TPIT-3** versus water fraction (*f_w_*) in water/tetrahydrofuran (THF) mixtures.

Fig. S18. Fluorescence emission spectra of **TPIT-5** versus water fraction (*f_w_*) in water/tetrahydrofuran (THF) mixtures.

Fig. S19. Fluorescence emission spectra of **TPIT-6** versus water fraction (*f_w_*) in water/tetrahydrofuran (THF) mixtures.

 Fig. S20. Fluorescent photographs of **TPIT-1** to **TPIT-6** powders under 365 nm excitation.

Fig. S21 Time-dependent changes in the fluorescence intensity of **TPIT-6 NPs** and **TPIT-6 NPs-Ab** (50 μM) incubated in PBS at 37℃.

Fig. S22 Cell viabilities of SMCs treated with different concentrations of **TPIT-6 NPs** for 24 hours. Error bars: standard deviation (SD), experimental times: n = 3.

Fig. S23 Cell viabilities of SMCs treated with different concentrations of **TPIT-6 NPs-Ab** for 24 hours. Error bars: standard deviation (SD), experimental times: n = 3.
